# Does Clean Energy Use Have Threshold Effects on Economic Development? A Case of Theoretical and Empirical Analyses from China

**DOI:** 10.3390/ijerph19159757

**Published:** 2022-08-08

**Authors:** Minglin Wang, Shaolong Zeng, Yunzhe Wang, Zhengxia He

**Affiliations:** School of Economics, Hangzhou Normal University, Hangzhou 311121, China

**Keywords:** clean energy, economic growth, threshold effects, panel threshold model, substitution effect

## Abstract

Increasingly serious energy security and environmental problems have become the main constraints to China’s economic development. Therefore, it is critical to explore the threshold effect of clean energy use on China’s economic growth. Based on the panel data of 30 Chinese provinces from 2000 to 2019 and using energy intensity (EI) as the threshold variable, this study adopts a panel threshold model to explore the threshold effect of clean energy development on the economy. Empirical results indicate that clean energy has a significant threshold effect on economic development, with the threshold value of EI being 0.7655. When EI is less than 0.7655, clean energy development has a more positive effect on economic growth. When the EI exceeds 0.7655, the impact is significantly positive but with a smaller coefficient. EI weakens the role of clean energy development in promoting economic growth. After 2015, the EI of most provinces in the sample was below the threshold value, which indicates that in recent years, with the economic cost of developing clean energy decreasing, the role of clean energy development in promoting the economy has become more significant. Therefore, we propose policy implications to better promote the effect of clean energy development in promoting economic growth.

## 1. Introduction

In October 2021, the Chinese government issued a document on achieving the goals of carbon peaking and neutrality based on the philosophy of new development. Five tasks are outlined in the document: slowing down carbon dioxide (CO_2_) emissions, enhancing energy efficiency, improving the proportion of non-fossil energy consumption, enhancing the carbon sink capacity of ecosystems, and building a green and low-carbon circular economy [1]. To achieve the dual goals of carbon peak and neutralization, the Chinese government introduced specific implementation plans to achieve peak CO_2_ emissions in key areas and sectors [2]. The carbon peaking and neutrality goals have caused China’s energy transformation to take a more active step. The vigorous development of China’s renewable energy has received greater support from the social and market development perspective. Many scholars believe that China’s clean energy industry ushers in a golden age of doubling in scale, such as moving from the current “small pond” to a broader “Pacific Ocean” [3,4,5,6,7].

Since the reform and opening up to the outside world (“since the reform and opening up” refers to “since December 1978”. Because in December 1978, at the Third Plenary Session of the 11th CPC Central Committee, the Chinese government decided that China begin to implement the policy of reform in the domestic country and opening up to the outside world), China’s clean energy industry has been booming, particularly photovoltaic and wind energy and the development of tidal power, ocean energy, geothermal power, biomass, and other energy sources has also been closely followed. With the joint promotion of vigorously developing new infrastructure, the renewable energy industry in China has achieved leapfrog development from weak to strong, and the total installed capacity of photovoltaic, hydropower, and wind energy has remained the highest in the world in the past five years. All countries currently regard clean energy development and transformation as the most important energy strategies.

On the one hand, actively developing clean energy can not only solve China’s increasingly serious energy security problems and effectively slow down the serious environmental problems caused by the consumption of nonrenewable energy but also help optimize the industrial structure and achieve the goal of green and low-carbon development. Simultaneously, it can promote the progress of green technology and expand employment. On the other hand, vigorously promoting clean energy research and development (R&D) requires not only large R&D funds and development talents but also large funds to establish corresponding supporting facilities.

There are many studies on the clean energy–economic development nexus in China, but the conclusions of the studies are inconsistent because of the differences in models, sample sizes, and empirical methods. In China, the correlation between the development of clean energy and economic growth remains insignificant. Additionally, it is worth confirming the role of clean energy development in driving economic development in a certain period. Based on annual panel data of 30 Chinese provinces from 2000 to 2019, this study applies the panel threshold empirical methodology and chooses energy intensity (EI) as the threshold variable to study the effect of clean energy development on economic growth in China. It was eventually found that EI weakened the influence of clean energy development on economic growth. Therefore, this paper intends to contribute to this field from the following aspects: (1) examine the threshold effect of the development of clean energy on economic growth and provide targeted policy implications for the government’s future resource allocation and energy structure adjustment, and (2) examine the regional heterogeneity manifested in the role of clean energy development on economic growth in China and provide a basis for the development of interprovincial cooperation in clean energy development.

The remainder of our paper is organized as follows: Section 2 is a literature review. Section 3 provides the theoretical analysis, including the theoretical mechanism analysis and assumption of the threshold effect. Section 4 describes the data source and empirical model construction. Section 5 provides an empirical analysis of the threshold role of clean energy development on regional economic growth, including empirical results and robustness tests. Section 6 presents the conclusions and Section 7 provides policy suggestions.

## 2. Literature Review

### 2.1. Energy–Economic Growth Nexus

Since the 1970s, the energy development–economic growth nexus has attracted the attention of academic and industry circles.

Based on the data from 1975 to 2001 in 18 developing countries, Lee examined the cointegration and causality between energy use and economic development and indicated that energy conservation negatively influenced economic development in these developing countries [8]. Guo used the Cobb–Douglas model and conducted an empirical analysis of the energy use–economic growth nexus in China from 1978 to 2016 and found a strong dependence of economic growth on energy [9]. Rahman explored the relationship among energy use, international trade, foreign direct investment (FDI), and economic development based on data from BRICS and ASEAN countries from 1990 to 2017. This study confirmed their long-term equilibrium interrelationship, and energy use was demonstrated to positively and significantly affect economic growth in these countries [10]. Doğanalp et al. explored the relationship between energy use, economic development, employment, and inflation using the panel vector autoregression method (PVAR), dynamic ordinary least squares (DOLS), and fully modified ordinary least squares (FMOLS) analysis in BRICS countries and found that energy use positively contributed to economic development [11].

Yang et al. indicated a long-run cointegration correlation and causality between coal consumption and economic growth in China [12,13]. Ma and Fu adopted a VAR model to explore whether there was a dynamic relationship between technological progress, energy use, and economic development. The results indicate a U-shaped relationship between energy use and economic development [14]. Duan and Si examined the relationship between carbon emissions, energy use, and economic development in China and proved a long-run equilibrium relationship among them [15]. Liang et al. found that the energy use–economic growth nexus in China showed a significant imbalance and asymmetry, and the influence of energy consumption on economic development showed threshold features [16]. Peng and Wu (2020) examined the long-term elasticity and causality between transportation sector energy consumption, economic growth, and CO_2_ emissions in China based on panel data of 30 provinces in China from 2004 to 2016. The results showed that all these variables had cointegration and two-way causality [17].

To achieve sustainable development, scholars have focused on the relationship between energy use and economic growth and have proposed four hypotheses [18,19,20]. The growth hypothesis argues that there is a unidirectional causality from energy use to economic growth and that energy is the essential input for economic development. The protection hypothesis argues that there is unidirectional causality from economic development to energy use but that there is no feedback. The feedback hypothesis posits a bidirectional causality between energy consumption and economic development, which has a two-way influence. The neutrality hypothesis does not hold for the causal relationship between the two because energy use is only a small part of the process of economic growth. The rationality of these hypotheses is affected by different factors, such as time length and energy category [19,20].

### 2.2. Environmental Kuznets Curve (EKC) and Energy–Economic Growth Nexus

The Environmental Kuznets curve (EKC) suggests that an inverted U-shaped curve exists between economic development and pollution. In the latest research, Wang et al. (2022) attempted to expand the EKC theory by considering urbanization and exploring the effect of urbanization on the emission–growth nexus. Based on data from 134 countries between 1996 and 2015, this study used urbanization as a threshold variable to explore the nonlinear influence mechanism of economic development on CO_2_ emissions and ecological footprint. The study showed that urbanization enhanced the positive impact of economic development on CO_2_ emissions and ecological footprint [21].

Because there existed a direct causality between energy use and environmental pollution, some scholars discussed the energy consumption–economic growth nexus based on the EKC hypothesis. Based on the EKC hypothesis, some studies have proposed the energy–environmental Kuznets curve (EyKC), which verified a nonlinear relationship between energy use and economic growth. Luzzati and Orsini (2009) examined the relationship between energy use and economic growth in 113 countries from 1971 to 2004. Studies have shown that, in many countries, there was no inverted U-shaped correlation between energy use and economic growth, and if the world was taken as a whole, the relationship between the two was monotonous and positively correlated, which did not support the EyKC hypothesis [22]. Liu et al., (2008) conducted a regression analysis on the relationship between per capita energy use, total energy use, and per capita gross domestic product (GDP) of 28 developed countries between 1970 and 2004 and found that the EyKC hypothesis only held in some countries, and there were other influencing factors affecting energy use [23]. Using the EyKC model, Zhao and Xue (2011) studied the dynamic correlation between energy use and economic growth from 1953 to 2008 and found that the relationship between energy use and economic growth in China first presented an inverted U-shaped and then a positive U-shaped (N-shaped) [24].

In summary, based on the EKC and the four hypotheses of energy use and economic growth, this study explores whether there is a nonlinear relationship between economic growth and the development of clean energy, which enriches studies on the interaction between the two.

### 2.3. Effect of Clean Energy Development on Economic Growth

With the vigorous development of the global clean energy industry, the effect of clean energy development on economic growth has increasingly attracted attention from academia and industry circles. Some scholars have examined the influence of the development of clean energy on economic growth [25,26,27]. Clean energy refers to energy that does not produce emissions in the process of energy production and consumption and causes less pollution to the environment than traditional energy sources. It includes wind, solar, biomass, nuclear, geothermal, hydropower, and ocean energy [8]. There are three main views on the effect of the development of clean energy on economic growth.

#### 2.3.1. Positive Effect

Many scholars have argued that clean energy development has positively affected economic growth [28,29,30,31,32,33,34,35,36,37,38,39].

Many studies have explored the different mechanisms through which that clean energy development significantly and positively affected economic development. Based on data from OECD countries, Awerbuch and Sauter indicated that clean energy development had important effects on economic development, energy security, and energy diversification [28]. Using panel data from Italy from 1997 to 2007, Magnani and Vaona concluded that renewable energy use positively influenced economic development at the local level in Italy and explained this positive influence as follows: clean energy use reduced the negative environmental externalities from fossil energy consumption [29]. Lin and Li constructed a research framework to explore the impact of clean energy policy on economic development in China. The results indicated that developing clean energy in the long term can promote economic structure upgrading in China and promote the sustainable economic development [30]. Zhang and Liu believed that clean energy development expedited capital and labor supplies, promoted energy-saving technological progress, and eventually led to accelerating long-term economic development [31]. Markandya et al. showed that the energy structure of the European Union (EU) had undergone major changes, from high-carbon energy to renewable energy, and had positively affected economic development [32].

In summary, although different studies adopted different sample periods, model methods, and types of clean energy, these studies argued that clean energy development positively influenced economic growth. Based on annual data from 85 economies from 1991 to 2012, Bhattacharya indicated that an increase in clean energy consumption significantly and positively influenced economic growth [33]. Okumus et al. indicated that clean energy development positively influenced economic growth in the Group of Seven (G7) countries [37]. Asiedu et al. examined the interrelationship between clean energy use, fossil energy use, carbon emissions, and economic development in European countries using data from the World Bank from 1990 to 2018. The results indicated a long-term interrelationship between fossil fuels, clean energy, CO_2_ emissions, and economic development [38]. Derrick et al. found that clean energy development had a major promoting effect on economic development in the short and long terms [39].

#### 2.3.2. Negative Effect

However, some studies have indicated that clean energy development negatively affected economic development [31,40,41,42,43,44,45,46].

Marques and Fuinhas indicated that the negative impact of clean energy development in the 24 European countries offset the positive impact of promoting economic development with the local development of natural resources; therefore, clean energy development did not seem to promote economic development [40]. Ocal and Aslan explored the correlation between clean energy use and economic growth and concluded that clean energy consumption negatively influenced economic development [41]. Zhang and Liu showed that if China gradually increased the proportion of clean energy to 35%, the resulting price rise would curb economic development in the short run [31]. Maji and Kabiru explored the effect of clean energy on economic development by adopting the autoregressive distributed lag (ARDL) methodology, and the long-term results showed a significant negative relationship between clean energy and economic development [42]. Mita et al. indicated that clean energy use significantly and negatively affected economic growth in Pakistan, Russia, and the United States [43]. Based on the PVAR model, Qi and Li explored the dynamic interrelationship between EU clean energy use and economic development, and the results indicated that clean energy consumption was conducive to emission abatement but at the expense of economic development in EU countries [44]. Pereira et al. examined the relationship between clean energy consumption and income, indicating either negative or non-significant short-term effects of clean energy use on economic development [46].

#### 2.3.3. Weak Influence

However, some studies have argued that renewable energy development has insignificant effects on regional economic development [47,48,49,50].

Employing data from 1949 to 2006 from the United States, Payne compared the causality between renewable and nonrenewable energy consumption and economic growth. The causality tests did not reveal a Granger causality between renewable or nonrenewable energy use and economic growth [47]. Based on data from 1997 to 2007 and adopting the random effects methodology, Menegaki examined the causality between economic development and clean energy development in 27 EU countries. The results did not show a causal relationship between clean energy use and economic growth. The estimated cointegration result indicated a weak relationship between economic development and clean energy consumption in the EU and suggested that the weak influence can be partly due to the unbalanced and insufficient development of clean energy in the EU [48]. Using data from 1990 to 2015 and employing cointegration and causality tests, Bulut and Muratoglu investigated whether clean energy use promoted economic growth in Turkey. The findings indicated the absence of causality between economic growth and clean energy use in Turkey [50].

### 2.4. Summary of the Literature

The study conclusions on clean energy development–economic growth nexus have not been consistent to date. Most studies have used panel data models to examine the effect of clean energy development on economic growth. Most of the studies collected data from the Organization for Economic Cooperation and Development (OECD) in developed countries, and few studies focused on other developing countries. In contrast, studies on China were even rarer [35,45,51]. Considering the vast territory of China and the significant differences between provinces in terms of economic and social development, government department management, and industrial structure, it is important to examine the regional heterogeneity of the role of clean energy development on economic growth in China based on Chinese provincial panel data.

The above literature is primarily based on a linear method to study the influence of clean energy development on economic growth. However, because clean energy development may affect economic growth through a variety of paths, there may be a nonlinear relationship between the two; therefore, the traditional linear method may not be accurate, leading to inconsistent conclusions. Therefore, as a supplement to the existing literature, and considering that EI can reflect the dependence of a country’s economy on energy, that is, the economic cost of developing clean energy, this paper intends to take EI as the threshold variable to study the nonlinear threshold effect of clean energy development on economic growth to provide relevant government departments with theoretical support and empirical basis to formulate targeted countermeasures for clean energy development based on the actual local situation.

## 3. Theoretical Mechanism Analysis and Assumption of Threshold Effect

According to the above literature, this paper provides the following theoretical mechanism analysis of the threshold effect, selects the threshold variable, and proposes an appropriate hypothesis before conducting the empirical analysis.

### 3.1. The Theoretical Mechanism Analysis of Threshold Effect

The EKC suggests an inverted U-shaped relationship between per capita income and environmental quality or pollution levels. As per capita income increases, environmental quality gradually deteriorates; however, when the economic development reaches a certain threshold, with the further increase in per capita income, people focus on environmental protection, and the government gradually adopts relevant policies to implement the environmental cost internalization. Simultaneously, the degree of environmental pollution gradually slows down, and the environmental quality is gradually enhanced. Based on the EKC hypothesis, this paper argues for the existence of a nonlinear relationship between economic growth and clean energy development, and EI can have threshold effects on this relationship.

The factor substitution effect between clean and nonrenewable energy shows an inverted U-shaped relationship, which is directly related to the replacement efficiency of clean energy. Therefore, when EI is less than a certain threshold, the cost of replacing nonrenewable energy with clean energy is lower, the replacement efficiency is higher, and the environmental pressure caused by nonrenewable energy can be reduced, which significantly and positively influences the macroeconomy. However, when the EI exceeds the threshold, the replacement cost of clean energy for nonrenewable energy increases because of the law of decreasing marginal technology replacement rate, and the marginal substitution effect is weakened, even making the marginal return of clean energy investment negative. In addition, the early stage of clean energy development requires a high initial fixed investment, and these investment costs should usually be paid by the government or the private sector. Because clean energy is often at a disadvantage in market competition under the current socialist market mechanism, government departments are required to implement more favorable subsidy and tax policies [52]. These subsidies for new energy sources have a crowding-out effect on other government expenditures [53]. In this case, the increased investment in clean energy occurs at the expense of economic development. Thus, there is a nonlinear relationship between economic development and clean energy development. A threshold model is selected to test this relationship.

### 3.2. Research Hypothesis

EI is usually used as a threshold variable, as in Chang et al., Qi and Li, and Huang et al. [45,46,49]. EI is calculated as the ratio of aggregate energy consumption to the real GDP. It is not difficult to see that it measures the degree of dependence on traditional energy in the process of economic development. When the EI value is low, clean energy replacement is more efficient and positively affects economic development. A higher EI value indicates a higher level of dependence when it is more economically costly to prioritize clean energy development and promote energy conversion. Simultaneously, it can be seen from the factor substitution effect between clean energy and fossil energy that when the EI value is higher, the difficulty and cost of factor substitution increase; therefore, EI may weaken the effect of the development of clean energy in promoting economic development. Therefore, we propose the following Hypothesis:

**Hypothesis:**  *There is a threshold effect of clean energy development in promoting regional economic growth, and EI is regarded as the threshold variable. That is, the effect of clean energy on driving economic growth weakens with an increase in EI.*

In summary, this study intends to select the annual panel data of 30 provinces in China from 2000 to 2019 and build a threshold regression model using the extended Cobb–Douglas production function. EI is regarded as the threshold variable to explore the threshold role of China’s clean energy development in promoting economic growth and provide scientific and reasonable guidance for the development of clean energy in China and the energy system transformation.

## 4. Data Sources and Empirical Models

### 4.1. Main Factors Affecting Economic Growth in the Cobb–Douglas Production Function

National economic growth generally refers to the growth in the total amount of products and services, that is, an increase in social property. GDP is the most important indicator system in the national economic accounting system and is widely used by governments worldwide to reflect national economic growth in a country and region. As the total amount of products and services produced by society increases, economic growth results from the comprehensive influence of various factors and is also restricted by various conditions. The main factors affecting economic growth in the traditional Cobb–Douglas production function include technological progress, capital investment, and human capital.

#### 4.1.1. Technological Progress

There is a close connection between technology and economic and social development. Without technology investment, productivity factors will not increase and will not effectively promote economic and social growth. Technology promotes economic growth in three ways. Technology improves the human factors in the production process, requires workers to have modern scientific knowledge and technical ability, and gradually changes from simple manual workers to mental workers. Technology has also improved the basic production factors in the production process and has promoted production technology innovation. In addition, technology clarifies new manufacturing mechanisms and provides new manufacturing technologies and methods, transforming them into new production relations.

#### 4.1.2. Capital Investment

Capital investment is the foundation of national economic growth. National economic development is due to the expansion of capital investment and production scale and the increase in social capital productivity to a certain extent. After capital input, in terms of supply, an increase in output is driven by an increase in product scale and productivity, resulting in an increase in total income. The increase in income leads to an improvement in the capacity of savings, which changes deposits into investment and causes investment growth. In terms of aggregate demand, increased income has led to increased demand for purchases and increased production capacity, which in turn has led to increased funding for investments. Therefore, mutual promotion between market supply and aggregate demand leads to a virtuous circle in national economic growth.

#### 4.1.3. Human Capital

Because of the differences in knowledge and technology of social personnel, labor factors in economic activities also have great differences, which affect productivity differences. Therefore, the development of the national economy needs to improve labor productivity further and increase the input of physical capital; however, it also depends on human social capital to a large extent; that is, the labor technology and product knowledge reserves reflected in the human body. Therefore, investment in social human capital, education and training of social workers, advancing the basic quality of social workers, and promoting the reserves of human social capital can improve labor productivity and drive national economic growth. Owing to national economic development, the demand for workers’ technical talents is diverse, which requires innovative scientific and technological talents and workers with professional skills to make the human capital investment reach a certain scale.

### 4.2. Constructing the Cobb–Douglas Production Function with Energy Elements

In recent years, climate change and low-carbon transformation have made the academic circles focus on the key role of clean energy in economic development. Clean energy, an independent production factor, was introduced into the extended Cobb–Douglas production function containing energy factors [25,26,27,54,55,56,57,58,59,60].

As China’s rural areas are still in the process of urbanization, rural urbanization not only causes a large surplus agricultural labor force to transition to city employment but also increases the accumulation of human resources and promotes further improvement in agricultural productivity. At present, China has entered the science and technology-driven stage; thus, the level of technological innovation ability and market openness significantly affects China’s economic development. Additionally, the reform and opening up to the outside world with the speeding up of China’s industrialization has improved China’s industrialization level and further promoted the rapid growth of China’s economy and society. Therefore, this study introduced clean energy development, urbanization level, level of scientific and technological innovation, level of industrialization, and degree of openness into the model. The model is given by Equation (1).

(1) $$Y_{it}=AK_{it}^{\alpha }L_{it}^{\beta }CE_{it}^{\beta_{1}}URB_{it}^{\beta_{2}}PAT_{it}^{\beta_{3}}lndu_{it}^{\beta_{4}}T_{it}^{\beta_{5}}$$

In Equation (1), the subscript *i* = 1, …, 30 represents the specific province, and the subscript *t* = 2000, …, 2019 represents the specific year; *Y*, *K*, *L*, *CE*, *URB*, *PAT*, *Indu,* and *T* represent output level, capital stock, labor stock, clean energy, urbanization level, scientific and technological innovation level, industrialization level, and transportation infrastructure, respectively. The production function is changed to Equation (2).

(2) $$\begin{array}{l} lnY_{it}=\beta_{0}+\beta_{1i}lnK_{it}+\beta_{2i}lnL_{it}+\beta_{3i}lnCE_{it}+\beta_{4i}lnURB_{it}+\beta_{5i}lnPAT_{it}\\ +\beta_{6i}lnlndu_{it}+\beta_{7i}lnT_{it}+\alpha_{i}+\nu_{t}+\varepsilon_{it} \end{array}$$

In Equation (2), *α*_*i*_ and *v*_*t*_ represent the unobservable individual fixed and time effect, $\varepsilon_{it}\sim iid\;N\left(0,\delta^{2}\right)$ is the random disturbance term.

### 4.3. Construction of Threshold Effects Model

To reduce the deviation caused by subjective division and correctly understand various factors affecting the impact direction and degree of clean energy development on economic growth. This study uses the panel threshold regression model provided by Dang et al. to endogenously classify scenarios based on the characteristics of the data to explore the heterogeneity of the effect of clean energy on economic development in each group [61]. The basic form of the constructed regression model is shown in Equation (3).

(3) $$\ln Y_{it}=\beta_{0}+\beta_{1i}\ln Y_{i,t-1}+\beta_{2i}\ln CE_{it}\cdot I\;\left(q_{it}\leq \gamma \right)+\beta_{3i}\ln CE_{it}\cdot I\;\left(q_{it}\geq \;\;\gamma \right)+\sum \beta_{ni}Z_{it}+\alpha_{i}+v_{t}+\varepsilon_{it}\;$$

In Equation (3), *lnCE_it_* is the core explanatory variable. The explanatory variables include *lnY*_*i,t−1*_ and *Z_it_*. *Z_it_* is a control variable matrix that has a significant influence on the actual output, except for clean energy. This includes capital stock, labor stock, urbanization level, scientific and technological innovation level, industrialization level, and transportation infrastructure. *β_ni_* is the coefficient. *q_it_* is the threshold variable, as selected by EI. For simplification, threshold variables are usually assumed to be smooth and exogenous. *γ* is a specific threshold value that divides a sample into two groups, and the coefficients of the two groups are *β_2i_* and *β_3i_*, respectively. *I (·)* represents an indicative function; and when the precondition is established, the value of the corresponding brackets is 1, and when the precondition is not established, the value is 0.

To test the statistical significance of the threshold effect, the *sup-Wald* test was adopted according to Hansen [62]. The null and alternative hypotheses were established for the test as below:

*H0: δ = 0*, any *γ ∈ Γ,*  *H1: δ ≠ 0*, part *γ ∈ Γ.* (4)

In Equation (4), $\Gamma =\left[\;\underline{\gamma }\;,\;\bar{\gamma }\;\right]$, $\underline{\gamma }$ and $\bar{\gamma }$ represent the two percentiles of the threshold variables. Assuming that the result completely rejects the null hypothesis, it proves a significant threshold effect. Thus, the following test statistic can be established:

(5) $$supW=supW_{n}(\gamma ),\;\;\gamma \in \Gamma$$

In Equation (5), for each fixed *γ*, *W_n_(γ)* is the standard Wald statistic:

(6) $$W_{n}(\gamma )=n\hat{\delta }(\gamma ){\hat{\sum_{\delta }\left(\gamma \right)}}^{-1}\hat{\delta }(\gamma )$$

In the Formula (6), $\hat{\delta }\left(\gamma \right)$ is the estimator of *δ* with fixed *γ*, and ${\hat{\sum_{\delta }\left(\gamma \right)}}^{-1}$ represents the consistent estimator of the asymptotic variance of $\hat{\delta }\left(\gamma \right)$.

Let $\left\{\begin{array}{l} G(\gamma )=(G_{\beta },{\;G}_{\delta }(\gamma ))\\ D(\gamma )=G{\left(\gamma \right)}^{T}\Omega^{-1}G(\gamma ) \end{array}\right.$.

Then the limit distribution of supW is

(7) $$supW\overset{d}{\to }supZ^{T}G{\left(\gamma \right)}^{T}D{\left(\gamma \right)}^{-1}R^{T}{\left[RD{\left(\gamma \right)}^{-1}R^{T}\right]}^{-1}RD{\left(\gamma \right)}^{-1}G(\gamma )Z,\;\gamma \in \Gamma \;$$

where *Z~N* (0, $\Omega^{-1}$). Based on the above statistics, the Bootstrap method was used [60,61], and the *p*-value and asymptotic distribution of the statistics were obtained. The decision rules are that the smaller the corresponding probability value, the more significant the threshold effect.

### 4.4. Related Variables and Data Sources

This paper selects the annual panel data of 30 provinces in China from 2000 to 2019. The 30 provinces covered in this paper are Hebei, Shanxi, Henan, Hubei, Hunan, Guangdong, Liaoning, Yunnan, Shaanxi, Gansu, Qinghai, Inner Mongolia, Ningxia Hui Autonomous Region, Jilin, Heilongjiang, Jiangsu, Zhejiang, Guangxi, Beijing, Anhui, Fujian, Jiangxi, Shandong, Hainan, Sichuan, Guizhou, Xinjiang Uygur Autonomous Region, Tianjin, Shanghai, and Chongqing. Because relevant data for Tibet, Hong Kong, Macao, and Taiwan are missing, the samples do not involve these four provinces.

#### 4.4.1. Explained Variable

The explained variable is the output level (Y, unit: CNY), and the actual per capita GDP in each province was used to reflect the economic growth. It was determined by real GDP per capita (2000 was taken as the constant price). GDP per capita was calculated by dividing the GDP published in the statistical yearbooks of China’s provinces by the national resident population.

#### 4.4.2. Explanatory Variables

The Cobb–Douglas production function contains the energy factors constructed above. The explanatory variables included the following production factors:

(1) Clean energy development (CE, unit: GW h). Because renewable energy re-accommodation was fully considered, and the clean energy industry development level may be underestimated through the evaluation of clean energy demand, the total output of clean energy was used to evaluate the development potential of clean energy and the scale of clean energy. Data were derived from the power output of primary energy in the energy balance sheet given by the Statistical Yearbook of Provinces and Cities and the clean energy power generation published by the China Energy Statistical Yearbook.
(2) Labor force (L) was measured by the proportion of employees with primary school, junior high school, senior high school, and college education or above published in the Statistical Yearbooks of Provinces and Cities for the year.
(3) Capital stock (K). This was reflected in the percentage of the balance of deposits and loans in the current year’s GDP published in the Statistical Yearbooks of Provinces and Cities.
(4) Urbanization level (URB) was reflected by the percentage of the urbanized population in the total population in the Statistical Yearbooks of Provinces and Cities.
(5) The level of scientific and technological innovation (PAT, unit: piece) was reflected in the number of patent authorizations published in the China Statistical Yearbook on Science and Technology.
(6) The industrialization level (Indu) was reflected in the percentage of industrial added value in the current year’s GDP published in the Statistical Yearbooks of Provinces and Cities.
(7) Transportation infrastructure (T) was reflected in the road distance per capita published in the Statistical Yearbook of Provinces and Cities.

### 4.5. Descriptive Statistics for Variables

Table 1 shows the descriptive statistics of each variable.

## 5. Empirical Analysis of the Threshold Effect of Clean Energy Development on Economic Growth

The empirical analysis of the threshold effect of the development of clean energy on regional economic growth was conducted from three perspectives. The first was to test the existence of the threshold effect, that is, to determine the statistical significance of the non-linear effect of clean energy development on regional economic growth. Specific threshold estimates could be obtained if there was a threshold effect. The second step was to judge whether the provinces were in the low- or high-threshold range, according to the threshold estimation. Finally, the coefficient of influence of clean energy development on regional economic growth was estimated within different threshold ranges.

### 5.1. Existence Test of Threshold Effect

It was essential to test for a threshold effect before using the panel threshold model for empirical analysis. This study adopted the bootstrap method to conduct the Wald test, and the null hypothesis was that there was ‘no threshold effect in the model’. However, based on the conclusions drawn after 500 repeated sampling, Table 2 indicates the threshold estimates and 95% confidence range of EI.

The results of the threshold effect test in Table 2 showed that the Wald statistic changed significantly at the 1% significance level, which means that the hypothesis of ‘no threshold effect’ is rejected. This means that clean energy development has a nonlinear threshold role in economic growth, and the threshold variable is EI. Based on the threshold estimates in Table 2, different EI values were divided into low and high EI intervals. The number of provinces with different EI intervals from 2000 to 2019 is listed in Table 3.

Table 3 shows the 30 provinces in which EI was higher than the threshold value in 2000 and 2005. After 2015, the EI of most provinces in the sample was lower than the threshold value.

### 5.2. Empirical Results of Panel Threshold Model

First, using the white test, the *p*-value was 0.00, which indicates heteroscedasticity in the model. Heteroscedasticity was then eliminated using the robust standard error regression method. A panel threshold model was adopted, and the threshold variable EI was selected for regression analysis. Table 4 presents the empirical results.

The results in Table 4 show that, when threshold variables were used, clean energy development had a significant threshold effect on economic growth. This proves the threshold effect of EI, and the threshold value was approximately 0.7655. When the EI was less than 0.7655, clean energy development positively influenced economic growth at the 1% significance level, and the influence coefficient was approximately 0.038. When EI exceeded 0.7655, the impact was significantly positive at the 10% significance level, and the influence coefficient was 0.021.

Based on the studies on the effects of energy dependence, a greater EI means that economic development depends more on fossil energy, and the economic cost of promoting clean energy development is higher. A significant negative correlation existed between EI and the substitution effect of clean energy. That is, a greater EI means a relatively weaker substitution effect, which means it is more difficult for clean energy to replace nonrenewable energy at this time. Meanwhile, the economic cost of developing clean energy is high. Thus, with the increase in EI in China’s provinces, the effect of clean energy development in promoting economic growth weakened; that is, EI weakens the effect of clean energy development in promoting economic growth. In other words, the economic cost of developing clean energy weakens the role of clean energy development in promoting economic growth. Our study has again verified that, whether EI is high or low, the transformation of the energy system from traditional to renewable energy is positively related to economic growth, similar to many studies [63]. Furthermore, our study shows that EI, that is, the economic cost of clean energy transformation, weakens the role of clean energy in promoting economic growth, which is also supported by current studies [45,55,64].

In addition, it is not difficult to draw from Table 3 that as of 2019, the EI of most provinces in the sample was lower than the threshold value, which means that clean energy development and promotion of energy transformation had a more positive influence on economic growth in most provinces in 2019.

### 5.3. Robustness Testing

Dai et al. argued that clean energy development is necessary for economic growth and a source of response to climate change using the energy mix as a measurement indicator for clean energy development [65]. We used the energy mix as the threshold variable to conduct this regression, which yielded insignificant results. The robustness of the panel threshold model was then tested by eliminating special samples that affected the conclusion. The method was to sort the share of clean energy production and then exclude the largest 5% and least 10% of the sample provinces, obtaining 27 and 24 sample provinces, respectively. This method reduced the adverse effects of extreme values. Panel data samples of 27 and 24 provinces in China were analyzed using the panel threshold model. Table 5 and Table 6 provide the regression prediction conclusions.

Table 5 and Table 6 show that when EI was less than the threshold value, the influence coefficient of clean energy development on economic growth remained positive at the 1% significance level, which shows that vigorously developing clean energy at this time still has significant and positive effects on economic growth. When EI exceeds the threshold value, the influence of clean energy on economic development is also significantly positive but with a smaller coefficient. This conclusion is consistent with the previous results in Table 4 from the significance level and the sign of the influence coefficient. This is verified again by the further robustness test showed in Table A1. Therefore, the test, after excluding the sample size, proves that the conclusion of this study is robust.

## 6. Conclusions

By selecting the panel data of China’s 30 provinces from 2000 to 2019, adopting the panel threshold model, and using EI as the threshold variable, we explored the threshold effect of clean energy development on economic growth. The main conclusions are as follows:

First, clean energy development has a significant threshold effect on economic growth. The value for the threshold variable, EI, was 0.7655. When EI was less than 0.7655, clean energy had a positive influence on economic growth at the 1% significance level, and the impact coefficient was 0.038. When EI exceeded 0.7655, the impact was also significantly positive but with a smaller coefficient (the influence coefficient was 0.021). Second, there is a significant negative correlation between EI and the substitution effect of clean energy. Therefore, a greater EI indicates a relatively weak substitution effect, which means that it is more difficult for clean energy to replace nonrenewable energy at this time, and the economic cost of developing clean energy is also high. In summary, when EI is high, clean energy development has a smaller positive influence on economic growth. When EI is low, economic growth is less dependent on energy; at this time, it is easy to transform into clean energy. The national economic level continues to improve owing to the improvement in energy utilization efficiency. Finally, as of 2019, the EI of each province in the sample was lower than the threshold value, which means that clean energy development and the promotion of energy transformation positively influence economic growth.

This study again confirmed that clean energy development significantly drives economic growth regardless of EI. However, for provinces with different EI ranges, the effect of the development of clean energy in promoting regional economic growth varies with the EI of different regions. For provinces that have crossed the threshold of EI, the transition to clean energy faces higher costs; therefore, in these provinces, clean energy development has little positive effect on regional economic growth. If we extend this conclusion to other countries in the world, we can conclude that for countries whose EI is lower than the threshold, the economic cost of developing clean energy is lower; therefore, the role of clean energy development in promoting economic growth in these countries is greater than those countries whose EI are higher than the threshold.

## 7. Policy Implications

The empirical results showed that after 2015, most provinces in the sample were generally in a low EI threshold range; therefore, promoting clean energy development will have a larger positive influence on economic growth. Currently, China is vigorously developing clean energy resources. It is necessary to focus on promoting interprovincial cooperation to enhance interprovincial technical exchanges and cooperation and deepen cooperation in energy conservation, emission reduction, and energy transformation to promote the continuous progress of regional economic integration. Simultaneously, each province should promote clean energy development according to local conditions.

### 7.1. Promoting Interprovincial Clean Energy Cooperation

First, local governments should take advantage of the resources in the region to create a national clean energy development demonstration base and promote the implementation of a number of clean energy demonstration projects, such as the national new energy microgrid, “photovoltaic+” cooperation mode, complementarity mode of water, wind, and light, and integrated renewable energy system solutions. The government should address the positive effect of renewable energy for maximizing energy conservation and consumption reduction and create a demonstration effect for the surrounding areas. Local governments in different provinces should implement formal communication mechanisms, break down information barriers, coordinate policies in different provinces, reduce redundant construction, avoid inefficient resource utilization, conduct comprehensive cooperation in clean energy development, and promote coordinated development among regions.

### 7.2. Develop Clean Energy according to Local Conditions

According to the above analysis, clean energy development has a threshold effect on economic growth in China. When EI is higher than the threshold value, there are higher economic costs to promote the development of clean energy. Therefore, targeted clean energy policies and objectives must be formulated according to regional differences when developing clean energy. The national policy on developing clean energy should be more inclined toward areas with higher economic costs of energy transition, such as areas with higher EI. For areas with a high economic cost of energy transition, that is, areas with higher EI than the threshold value in this study, when developing clean energy, it is necessary to explore a characteristic path suitable for clean energy development in the region. Simultaneously, China should focus on areas with high EI and create more favorable subsidy and tax policies for energy transition in these regions.

### 7.3. Strengthening Technological Innovation and International Cooperation

Clean energy development is inseparable from consolidating its foundation, which promotes the continuous updating of its technology. Clean energy is a high-cost industry. To reduce costs, a company must improve its innovation ability and scale. In addition, as an industry grows, the government must play a leading role in R&D and stimulate people’s interest in R&D through support and encouragement. At the same time, it is necessary to strengthen the independent innovation ability of small- and medium-sized companies, improve the protection of public intellectual property rights, and actively create an atmosphere of an innovative society. Concurrently, owing to a country’s limited technical level and scope, international cooperation can enable the development of clean energy projects to master more innovative and core technologies, which greatly improves the development speed of clean energy. This is important for efficient, clean energy development. For example, the government can send excellent workers or researchers to countries with excellent clean energy development for training and education or are committed to building an international platform for mutual benefit, close cooperation, and common development.

Although this study considers EI as the threshold variable to propose new insights into the nonlinear threshold effect of clean energy development driving China’s regional economic growth, the selection of relevant control variables remains incomprehensive; for example, the impact of economic openness, foreign trade, and foreign direct investment on economic growth has not been considered. Future studies should consider including more control variables to obtain a more comprehensive view or changing the threshold variables to examine the nonlinear influence of clean energy development on economic growth from another perspective.

## Figures and Tables

**Table 1:** Descriptive statistics of variables.

Variable	Mean	Std. Dev.	Minimum	Maximum	Observations
lnY	9.907	0.736	7.959	11.750	600
lnCE	4.333	2.554	−4.605	8.136	600
lnK	2.853	1.188	1.347	8.725	600
lnL	9.248	1.349	5.975	13.828	600
lnURB	3.838	0.358	2.671	4.495	600
lnPAT	8.875	1.760	3.466	13.180	600
lnInd	3.480	0.575	0.446	5.406	600
T	3.176	0.687	0.989	4.956	600
EI	1.231	0.833	0.208	7.962	600

**Table 2:** Threshold effect test results.

Threshold Variable	Threshold Estimators	F-Value	*p*-Value
EI	0.7655 **	41.94	0.0038

Note: ** denotes rejection of the hypothesis at a 0.05 significance level.

**Table 3:** Number of provinces in different EI intervals, 2000–2019.

Threshold Intervals	2000	2005	2010	2015	2019
EI ≤ γ	0	0	9	22	21
EI ≥ γ	30	30	21	8	9

**Table 4:** Estimation results of the panel threshold model.

Variable	Coef.	t
$\ln{\mathrm{CE}}_{\mathrm{it}}\cdot I\;\left(q_{\mathrm{it}}\leq \gamma \right)$	0.038 ***	2.96
$\ln{\mathrm{CE}}_{\mathrm{it}}\cdot I\;\left(q_{\mathrm{it}}\geq \gamma \right)$	0.021 *	1.90
lnK	0.071 **	2.64
lnL	0.184 ***	8.74
lnPAT	0.095 ***	4.67
lnURB	0.319 ***	3.38
lnIndu	0.180 ***	2.89
T	0.355 ***	6.65
Constant	4.052 ***	12.14
Observations	600	/

Notes: *** denotes rejection of the hypothesis at a 0.01 significance level. ** denotes rejection of the hypothesis at a 0.05 significance level. * denotes rejection of the hypothesis at a 0.1 significance level.

**Table 5:** Regression results of 27 provinces after excluding special samples.

Variable	Coef.	t-Statistic
$\ln{\mathrm{CE}}_{\mathrm{it}}\cdot I\;\left(q_{\mathrm{it}}\leq \gamma \right)$	0.036 ***	3.03
$\ln{\mathrm{CE}}_{\mathrm{it}}\cdot I\;\left(q_{\mathrm{it}}\geq \gamma \right)$	0.018 *	1.72
lnK	0.061 **	2.34
lnL	0.189 ***	10.20
lnPAT	0.092 ***	4.83
lnURB	0.395 ***	4.86
lnIndu	0.180 ***	2.81
T	0.333 ***	6.40
Constant	3.872 ***	11.92
Observations	540	/

Notes: *** denotes rejection of the hypothesis at a 0.01 significance level. ** denotes rejection of the hypothesis at a 0.05 significance level. * denotes rejection of the hypothesis at a 0.1 significance level.

**Table 6:** Regression results of 24 provinces after excluding special samples.

Variable	Coefficient	t-Statistic
$\ln{\mathrm{CE}}_{\mathrm{it}}\cdot I\;\left(q_{\mathrm{it}}\leq \gamma \right)$	0.038 ***	3.21
$\ln{\mathrm{CE}}_{\mathrm{it}}\cdot I\;\left(q_{\mathrm{it}}\geq \gamma \right)$	0.019 *	1.76
lnK	0.057 **	2.09
lnL	0.190 ***	9.66
lnPAT	0.088 ***	4.79
lnURB	0.374 ***	3.87
lnIndu	0.174 **	2.70
T	0.350 ***	6.04
Constant	3.953 ***	10.72
Observations	480	/

Notes: *** denotes rejection of the hypothesis at a 0.01 significance level. ** denotes rejection of the hypothesis at a 0.05 significance level. * denotes rejection of the hypothesis at a 0.1 significance level.

## Abbreviations

**Abbreviations**	**Key Term **
EI	energy intensity
CO_2_	carbon dioxide
R&D	research and development
FDI	foreign direct investment
PVAR	panel vector autoregression
DOLS	dynamic ordinary least squares
FMOLS	fully modified ordinary least squares
EKC	Environmental Kuznets curve
EyKC	Energy–environmental Kuznets curve
GDP	gross domestic product
EU	European Union
ARDL	autoregressive distributed lag
OECD	Organization for Economic Cooperation and Development
CE	Clean energy development
L	Labor force
K	Capital stock
URB	Urbanization level
PAT	The level of scientific and technological innovation
Indu	The industrialization level
T	Transportation infrastructure

## Appendix A

In a further robustness test, EI was replaced by the energy consumption published in China’s energy statistical yearbook divided by the total industrial output value (EI2). We used EI2 as the threshold variable to make the robustness testing for the following reason: as a large developing country, China’s rapid economic growth is basically supported by the high consumption of energy minerals such as coal, oil, and natural gas in the process of industrialization. The change in energy consumption profoundly reflects the industrial development of a country, and there is a strong relationship between energy consumption and industrial output value. We used EI2 as the threshold variable to make the regression to verify the threshold effect of clean energy development on economic growth. The results were as follows:

**Table A1:** Robustness Testing Results using EI2 as the threshold variable.

Variable	Coefficient	t-Statistic
$\ln{\mathrm{CE}}_{\mathrm{it}}\cdot I\;\left(q_{\mathrm{it}}\leq \gamma \right)$	0.039 **	3.45
$\ln{\mathrm{CE}}_{\mathrm{it}}\cdot I\;\left(q_{\mathrm{it}}\geq \gamma \right)$	0.018 *	1.72
lnK	0.065 **	2.55
lnL	0.184 ***	10.38
lnPAT	0.096 ***	5.11
lnURB	0.352 ***	4.07
lnIndu	0.186 ***	3.09
T	0.340 ***	6.34
Constant	3.967 ***	12.80
Observations	600	/

Notes: *** denotes rejection of the hypothesis at a 0.01 significance level. ** denotes rejection of the hypothesis at a 0.05 significance level. * denotes rejection of the hypothesis at a 0.1 significance level.

This result was basically consistent with our results in Table 4, in which EI was used as the threshold variable. This verified again that EI as the measurement for the difficulty and cost of developing clean energy weakens the role of clean energy development in promoting economic growth.
